# Protocol for the generation of Symbiodiniaceae mutants using UV mutagenesis

**DOI:** 10.1016/j.xpro.2023.102627

**Published:** 2023-10-03

**Authors:** Joseph A. Russo, Tingting Xiang, Robert E. Jinkerson

**Affiliations:** 1 Department of Chemical and Environmental Engineering, University of California, Riverside, Riverside, CA 92521, USA; 2 Department of Microbiology, University of California, Riverside, Riverside, CA 92521, USA; 3 Center for Plant Cell Biology, Department of Botany and Plant Sciences, University of California, Riverside, Riverside, CA 92521, USA; 4 Department of Bioengineering, University of California, Riverside, Riverside, CA 92521, USA

**Keywords:** Genetics, Microbiology, Model Organisms, Environmental sciences

## Abstract

Genetic approaches are limited in the dinoflagellate family, Symbiodiniaceae, causing a bottleneck in the discovery of useful mutants toward the goal of preventing future coral bleaching events. In this protocol, we demonstrate the application of UV exposure, coupled with downstream phenotypic screening and mutant isolation, to form a UV mutagenesis pipeline. This pipeline provides an avenue to generate Symbiodiniaceae mutants to help link genotype to phenotype, as well as address previously unanswered questions surrounding relationships with host organisms, like coral.

For complete details on the use and execution of this protocol, please refer to Jinkerson et al. (2022).^1^

## Highlights

• Generate growth curves of a chosen Symbiodiniaceae strain and scale-up cultures
• Create a survival curve of the chosen Symbiodiniaceae strain in response to UV exposure
• Discover mutants in Symbiodiniaceae through downstream screening processes

## Abstract (editor highlights)

Publisher’s note: Undertaking any experimental protocol requires adherence to local institutional guidelines for laboratory safety and ethics.

## Graphical abstract

**Figure:**
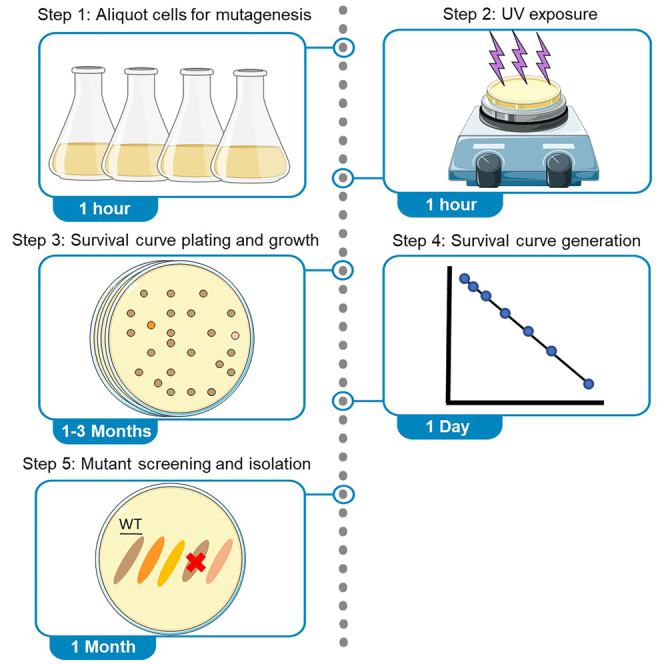

## Before you begin

Symbiodiniaceae are a family of marine dinoflagellates that are morphologically characterized by their brown color and thecal plate cell walls.^2^^,^^3^ They are also well known for complex genetic features such as novel DNA packaging proteins, and unique gene organization.^4^^,^^5^^,^^6^ Although Symbiodiniaceae can be free living, many strains participate in symbiotic relationships with host species. They can form associations with host organisms like foraminifera, clams, ciliates, and flatworms, but are most known for associating with corals and other species within the phylum Cnidaria.^2^ They provide photosynthetically produced carbohydrates in exchange for sources of nitrogen and other nutrients.^7^^,^^8^

Coral-Symbiodiniaceae symbioses are ecologically and economically important. They support coral reef ecosystems and industries, like fisheries.^9^ However, these ecosystems are threatened by coral bleaching phenomena, the loss of algae from the host, that occur in response to environmental stresses. Prolonged environmental stress and the absence of associated algae to provide carbohydrates can result in the death of the host.^10^ Despite the importance of understanding this relationship, there are many gaps in knowledge about how these organisms interact and how specific algal genotypes and phenotypes affect the symbiosis.

Progress towards the creation of stable Symbiodiniaceae mutants has been limited due to difficulties with direct genetic engineering techniques.^4^^,^^5^ This is thought to be related to unique features of dinoflagellates like the dinokaryon nuclei, a structure containing permanently condensed chromosomes in a liquid crystalline state, and non-classical histone-like proteins.^4^^,^^5^^,^^6^ The utilization of UV mutagenesis coupled with phenotypic screening is intended to overcome these limitations by circumventing the reliance on molecular tools to create mutations and produce strains with phenotypes of interest.

This UV mutagenesis approach was performed with *Breviolum minutum* but is likely applicable to other axenically cultured Symbiodiniaceae species. Axenic cultures are necessary to allow Symbiodiniaceae colony growth on solid media without competition from bacteria and fungi. Several features of Symbiodiniaceae enable mutant identification using the UV mutagenesis approach: (1) a haploid genome, which allows creation of homozygous mutations from the UV damage;^11^^,^^12^^,^^13^ (2) the ability to form clonal colonies on agar plates; and (3) the ability to visually screen and identify mutant color phenotypes from the colonies. Furthermore, the ability of *Breviolum minutum* to grow both photo-autotrophically in the light and heterotrophically in the dark supplied with glucose as a carbon and energy source,^1^^,^^14^ has allowed for the creation of mutants deficient in photosynthesis, showing the utility of this approach.^1^

### Preparation of sterile liquid marine Broth (MB) media

**Timing: 2 h**

1. Add 37.4 g MB media mix per liter of ultrapure water.
2. Autoclave the solution at 121°C for 30 min.
3. Remove from the autoclave and allow to cool to 22°C.

***Note:*** Small particles of precipitation in liquid MB medium will accumulate at the bottom of the bottle after autoclaving and can make the media cloudy if disturbed during use. These particles should be avoided and may affect the ability of the UV radiation to reach the cells, reducing efficacy of mutagenesis. These small, precipitated particles can be filtered out of the media into a new sterile bottle using a sterile vacuum 0.22-micron filtration system.

### Preparation of physiologically consistent Symbiodiniaceae cultures

**Timing: ∼3 months**

Healthy, axenic cultures are important to ensure the quality of colonies produced downstream and isolate mutant colonies of interest. Multiple replicate flasks are created to ensure the availability of cells. This protocol is performed using axenic cultures of *Breviolum minutum*, but other axenic Symbiodiniaceae isolates can be substituted. If an axenic culture of the Symbiodiniaceae strain of interest does not yet exist, isolation can be attempted using the approach outlined in Xiang et al., 2013, and detailed in the Xiang and Grossman, 2018 protocol.^15^

4. Prepare an ideal growth environment.
  a. Set incubator settings to achieve a temperature of 27°C and 12 h-light/12 h-dark light cycle with a light intensity of 5–10 μmol photons m^−2^ s^−1^ of PAR.
5. Prepare liquid pre-cultures of Symbiodiniaceae.
  a. Obtain an axenic culture of Symbiodiniaceae on solid media.
  b. Fill a 50 mL flask with 25 mL of liquid MB media.
  c. Inoculate the MB media with axenic Symbiodiniaceae cells, from a single colony on an agar plate.
  d. Repeat this process to generate a minimum of 3 replicate pre-culture flasks.
  e. Place cultures into the growth environment.
  f. Allow the inoculated culture to grow for approximately 2–3 weeks or reach a cell density of approximately 1 × 10^6^ cells per mL before use.

***Note:*** Bacterial or fungal contamination in liquid media manifests as opaque liquid or clumps of material. Contamination on solid media can be visually distinguished by the differences in colony morphology.

6. Generate growth curve.
  a. Aliquot 1.5 × 10^7^ cells from each preculture flask into a sterile 50 mL centrifuge tube from the prepared pre-culture flask.
  b. Centrifuge the aliquot for 5 min at 500 × *g* and resuspend cells in 1 mL of sterile MB.
  c. Add 74 mL of sterile MB and 1 mL cell solution to a sterilized 250 mL flask to reach a final concentration of 2 × 10^5^ cells/mL.
  d. Repeat this process to generate a minimum of 3 replicate growth curve flasks.
  e. Collect cell concentration data every 4–5 days to monitor the growth of the culture. Note when the culture reaches lag, log, and stationary phases as demonstrated by the diagram in Figure 1.

**Figure 1:**
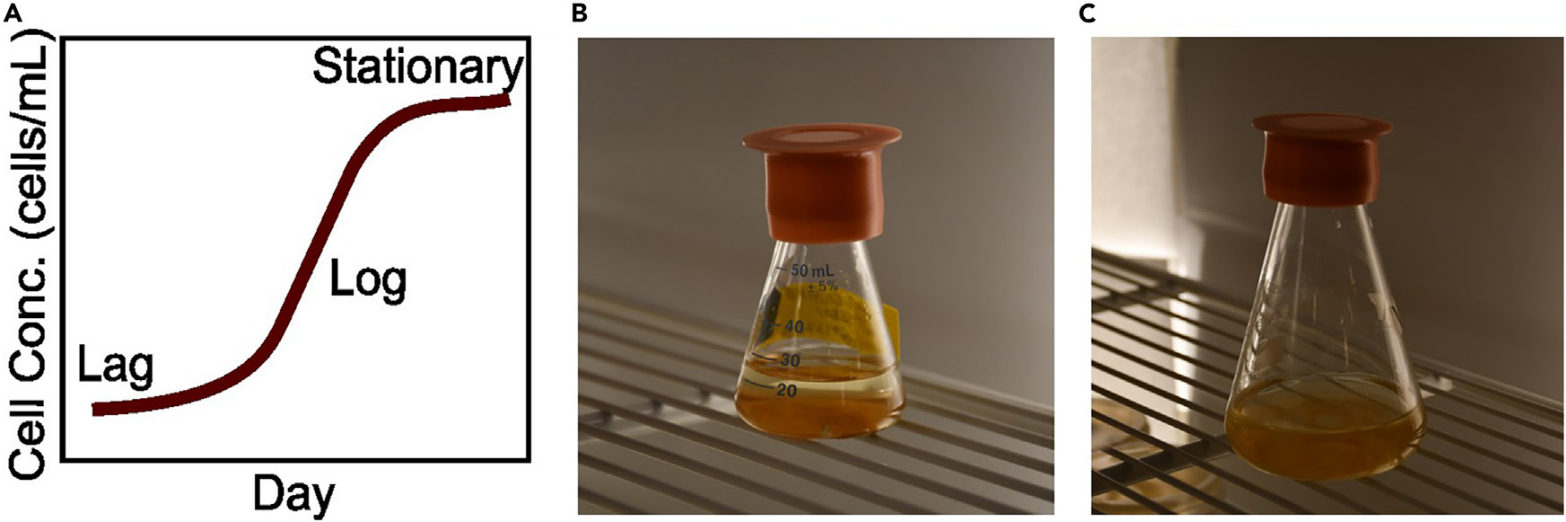
Culture preparation and growth environment (A) Growth curve diagram to identify stages of a growth curve. (B and C) (B) A 50 mL flask containing Symbiodiniaceae cells in MB-based media, inoculated from axenic culture plates, and allowed to grow and (C) a 250 mL flask containing Symbiodiniaceae culture at a concentration of 1 × 10^6^ cells/mL in MB-based media.
  f. Stop data collection after the culture enters stationary phase, indicated by no change in cell concentration for at least two time points in a row.
7. Scale up culture.
  a. Repeat Step 4 to create 3 pre-cultures cultures with a minimum concentration of 1 × 10^6^ cells/ mL.
  b. From each pre-culture, aliquot 1.5 × 10^7^ cells into a 50 mL microcentrifuge tube, centrifuge and resuspend the cells in 5 mL of fresh MB media, and transfer the cells to a new, sterilized 250 mL flask containing 70 mL of MB, to create a culture with a final concentration of 2 × 10^5^ cells/mL.
  c. Allow the cultures to grow to a concentration of 1 × 10^6^ cells/mL.
  d. For each culture, propagate by repeating the process in step 7b.
  e. Allow the culture to grow into log phase and a concentration of at least 2 × 10^6^ cells/mL before use in the mutagenesis procedure.

### Preparation of marine Broth (MB) supplemented with 0.5 g/L glucose solid media

**Timing: ∼3 h**

8. Prepare solid MB supplemented with 0.5 g/L glucose media (1 L).
  a. Add MB media mix, glucose, and agar (1.5% w/v), with Ultrapure water, to a desired volume and autoclave 121°C for 30 min. Approximately 2000 mL minimum will be required.
  b. Allow the medium to cool slightly to 60°C–70°C.
  c. Transfer media to 100 mm × 15 mm plastic Petri dishes using a serological pipette at a volume of 24 mL per dish until all prepare media is used. Avoid disturbing and/or transferring any precipitated particles that may be present in the bottle.
  d. Turn off the biosafety cabinet and let the agar media filled Petri dishes dry for 12 h inside of the cabinet with the sash down.

### Preparation of sterile equipment for mutagenesis

**Timing: ∼2 h**

9. Place a large metal paper clip (approximately 47 mm in length) on the inside of each empty glass Petri dish (7 total, or as many needed for each time point) and wrap the Petri dishes in aluminum foil for autoclaving.
10. Autoclave at 121°C for 30 min.

## Key resources table

**Table:** 

REAGENT or RESOURCE	SOURCE	IDENTIFIER
**Chemicals, peptides, and recombinant proteins**		

BD Difco marine broth 2216	BD Industrial/Difco	Cat# DF0791-17-4
Dextrose/D-glucose	Fisher Chemical	Cat# D16-3
Agar	Fisher BioReagents	Cat# BP1423-500

**Experimental models: Organisms/strains**		

Clonal axenic *Breviolum minutum* (clade B) strain SSB01	The Aiptasia Symbiosis Resource	SSB01

**Software and algorithms**		

Excel	Microsoft Corporation	https://www.microsoft.com/

**Other**		

Glass Petri dishes (100 mm × 15 mm)	Pyrex	Cat# 08-747C
1-⅞ jumbo smooth paper clip	Office Depot	Cat# 10004
UV lamp	NuAire	Cat# X-998442-01
Petri dishes with clear lid	Fisher	Cat# S33580A
Borosilicate glass disposable serological pipets with regular tip, standard length	Fisher	Cat# 13-678-25E
Easypet 3 Electronic Pipet Filler and Controller	Eppendorf	Cat# 4430000018
Isotemp 4 × 4 ceramic stir plate	Fisher Scientific	Cat# 11-100-16S
TipOne 1–200 μl natural, beveled pipet tips in sterilized stacks, 5 layers	USA Scientific	Cat# 1111-0200
Serological pipets, 1 mL	Thermo Scientific	Cat# 170353N
PIPETMAN L P200L, 20–200 μL, metal ejector	Gilson	Cat# FA10005M
Round wood toothpicks	N/A	N/A
Stand for stir plate	N/A	N/A
Dissection stereoscope	Olympus	Cat# SZ51
Disposable cell spreader	Excel Scientific	Cat# 470206-444
Class II type A2 biosafety cabinet	NuAire	Cat# NU-425-400
Incubator/growth chamber	Percival	Cat# I-36VL
Erlenmeyer flask, 50 mL	Eisco	Cat# S23898
Erlenmeyer flask, 250 mL	Fisherbrand	Cat# FB500250
Silicone sponge closure, 20 mm	Chemglass	Cat# CLS-1490-020
Silicone sponge closure, 28 mm	Chemglass	Cat# CLS-1490-028
50 mL centrifuge tube	VWR	Cat# 10025-696
Serological pipets, 25 mL	VWR	Cat# 75816-090

## Materials and equipment

Materials and reagents that touch the cell culture must be sterile with sterility maintained throughout the process.

**Table:** Liquid MB Media

Reagent	Final concentration	Amount
BD Difco Marine Broth 2216	37.4 g/L	37.4 g
Ultrapure Water	N/A	1 L

Store at 22°C, for a maximum of 1 year.

**Table:** Solid MB + Glucose Media

Reagent	Final concentration	Amount
BD Difco Marine Broth 2216	37.4 g/L	37.4 g
Glucose	0.5 g/L	0.5 g
Ultrapure water	N/A	1 L
Agar	15 g/L	15 g

Store at 22°C, for a maximum of 1 year.

## Step-by-step method details

### Aliquot cells for mutagenesis

**Timing: 1 h**

This step outlines the preparation of cell solutions (an “aliquot”) for each UV exposure period included in the survival curve. Seven exposure periods have been previously used and are included in the outline provided; however, the duration of the exposure periods and number of exposure periods can be modified if needed. The cell concentration of each culture is standardized to ensure consistent exposure to UV radiation. Once the survival curve is established, aliquots can be made as needed for UV exposure at a chosen exposure period.

1. Choose a pre-cultured 250 mL flask with physiologically consistent cells and minimum concentration of 2 × 10^6^ cells/mL.
2. Transfer cells to 50 mL centrifuge tubes for each planned time exposure.
  a. Obtain cell concentration of axenic cell cultures in log phase.
  b. Calculate volume of culture required to transfer a total of 2 × 10^7^ cells.
  c. Transfer calculated volume of cell culture to 50 mL tubes for each time point.
3. Adjust concentration of aliquoted solution to 1 × 10^6^ cells/mL.
  a. Centrifuge transferred cells and remove supernatant.
  b. Resuspend cells in a final volume 20 mL of fresh MB media.
4. Set aside tubes for UV exposure.

### UV exposure

**Timing: 1 h**

Each cell aliquot prepared in the previous step will be exposed to germicidal UV light for one of the following periods of time: 0, 15, 30, 60, 120, 180, and 240 s. The time periods of UV exposure given here have been chosen because of their previous success with *Breviolum minutum.* This procedure should be performed inside a biological safety cabinet or flow hood. Glass materials, such as serological pipettes and Petri dishes, should also be sterilized prior to use and have been chosen for use to ensure the efficient retrieval of mutagenized cells after UV exposure.

**Figure 2:**
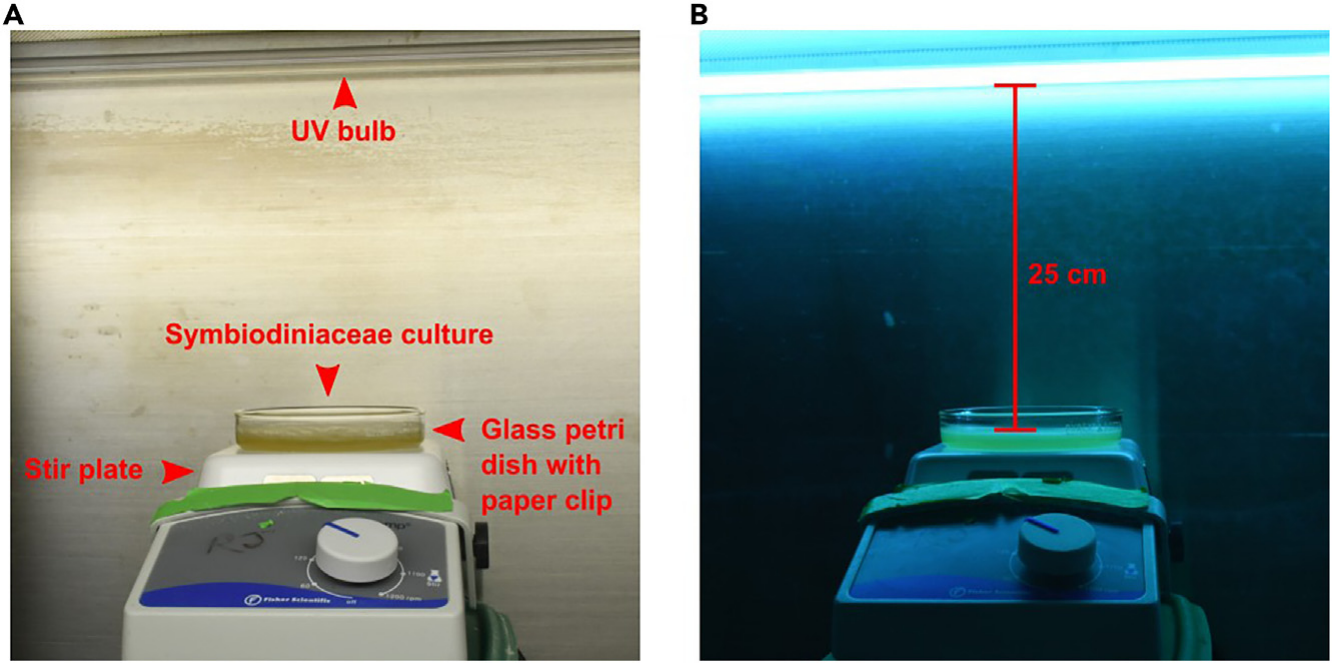
UV exposure environment (A) *Breviolum minutum* culture before UV exposure. (B) *Breviolum minutum* culture during UV exposure. Red line indicates the 25 cm distance from the bulb to the culture.

***Note:*** Take precautions to ensure safety from UV exposure by closing the sash or protective covering of the hood and/or leaving the area.

5. Prepare a sterile environment for UV exposure as demonstrated in Figure 2.
  a. Place the stir plate 25 cm from the UV bulb within the biological safety cabinet. A stand may be required to achieve this distance. Disinfect each item with 70% ethanol.
  b. Place glass Petri dishes and serological pipettes into the biosafety cabinet. Disinfect each item with 70% ethanol.
  c. Turn on the UV lamp before starting the aliquoting procedure for a minimum of 15 min, to further disinfect materials transferred into the biological safety cabinet.

6. Transfer aliquot for UV exposure.
  a. Using a sterile glass serological pipette, mix and transfer the entirety of a chosen cell aliquot with a chosen exposure period to the bottom half of a sterile glass Petri dish containing a metal paper clip.
  b. Place the dish on top of the stir plate.
  c. Allow the culture to mix at a rate of 2–3 revolutions per second, ensuring that the cell solution is homogeneous and does not leave the inside of the Petri dish.
7. Perform UV exposure.
  a. Turn on the UV bulb for the intended time exposure period as shown in Figure 2B.

***Note:*** Homogeneity of the cell culture during UV exposure is important to ensure even exposure to all the cells in solution.

8. Transfer mutagenized cells to a new 50 mL centrifuge tube using a new sterile glass serological pipette.

***Note:*** Cells are more likely to stick to the surface of the Petri dish and pipette after being mutagenized. Additional fresh media can be used to remove cells that are adhering to the Petri dish if necessary. The cell solution can then be centrifuged resuspended to the appropriate cell concentration to account for the added media.

Repeat steps 6–8 for each exposure period: 0, 15, 30, 60, 120, 180, 240 s.

**Pause point:** After UV exposure the cells can be kept for 12–24 hours, in their respective tubes, for plating the next day if needed.

### Survival curve plating and growth

**Timing: 1–3 months**

Perform a serial dilution of the mutagenized cells from each time point. In a sterile environment, a small volume of dilutions containing 1 × 10^5^, 1 × 10^4^, or 1 × 10^3^ mutagenized cells will be plated in parallel. The numbers stated here reflect the number of mutagenized cells to be transferred; only a fraction will survive the mutagenesis process and form colonies on the plate. Plating a range of cell concentrations ensures the ability to quantify colonies, as too many colonies may increase the difficulty of counting the number accurately.

9. Prepare a serial dilution of mutagenized cells for plating.
  a. Centrifuge and resuspend the mutagenized cell cultures of each time exposure period to a concentration of 1 × 10^6^ cells/mL in fresh MB.
  b. From each tube, transfer 1 mL of each 1 × 10^6^ cells/mL dilution into a 15 mL centrifuge tube containing 9 mL of fresh MB to obtain a concentration of 1 × 10^5^ cells per mL.
  c. Transfer 1 mL of each 1 × 10^5^ cells per mL solution to a new tube containing 9 mL of fresh MB to obtain a concentration of 1 × 10^4^ cells per mL.
10. Plating of mutagenized cells.
  a. Label previously prepared agar media plates for each mutagenized cell dilution created in the previous step. Include the number of cells added, UV exposure period, and replicate name (“r1”, etc.).
  b. Using 1 mL serological pipette, transfer a 100 μL aliquot from the prepared 1 × 10^6^ cells per mL solution onto at least 3 dishes of 0.5 g/L glucose + MB media, close the dish lid, and set the dish to the side.
  c. Evenly spread the mutagenized cell solution across the Petri dish using a new disposable spreader for each plate.
  d. Allow each plate to dry thoroughly with the lid off within the sterile hood and then parafilm. Each plate will require approximately 2–3 min to dry.
  e. Repeat this process with the 1 × 10^5^ and 1 × 10^4^ cells per mL solutions.

***Note:*** A consistent volume and mixing of the solution during plating is important to ensure comparable numbers across replicates.

***Note:*** Ensure plates are thoroughly dried before sealing with parafilm. Any residual liquid may move within the Petri dish and spread colony cells after the initial plating date. This may cause over-counting errors, affecting accuracy.

11. Place Petri dishes with mutagenized cells into the growth environment prepared for physiologically consistent Symbiodiniaceae as demonstrated in Figure 3A.

**Figure 3:**
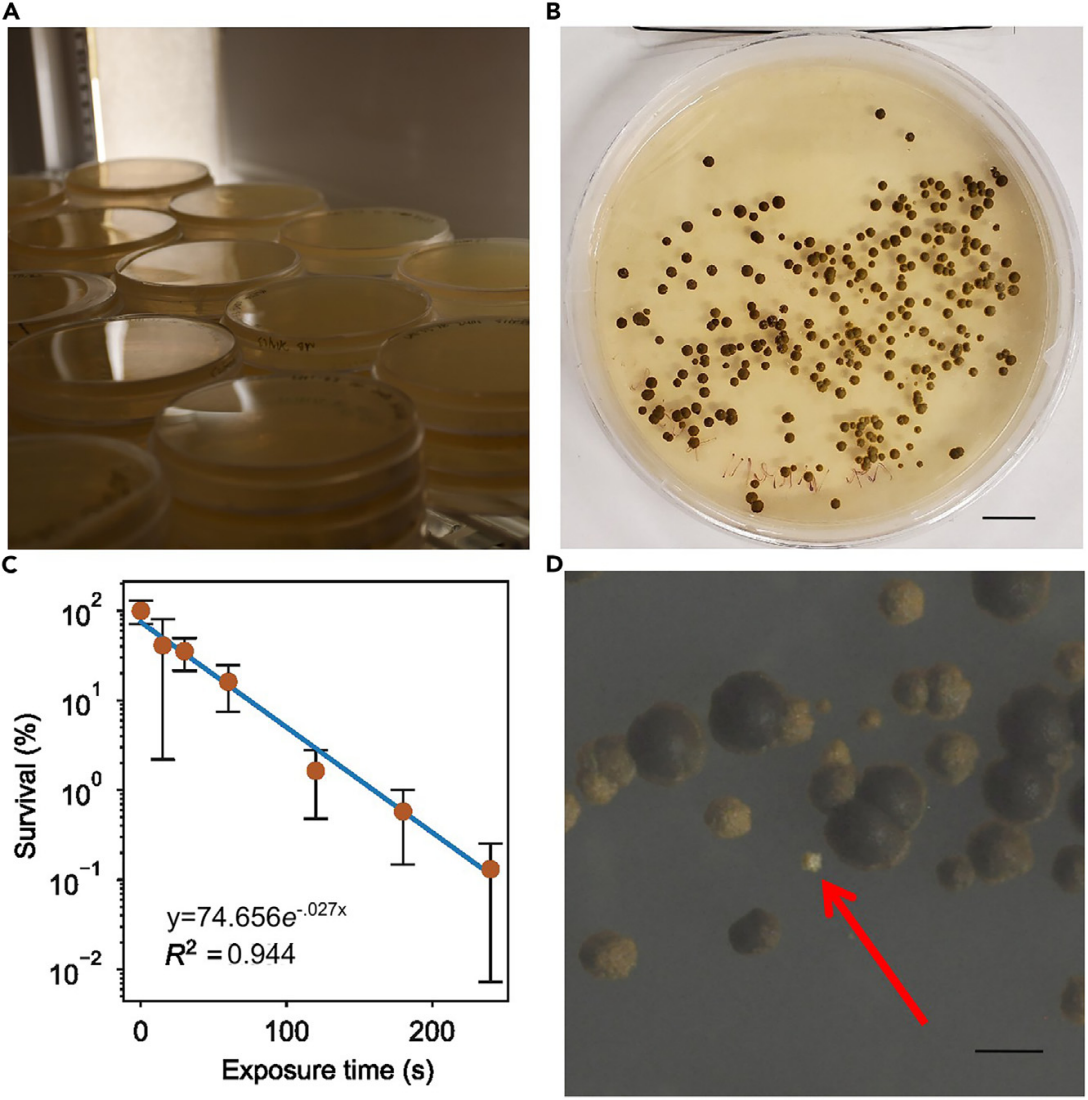
Survival curve and growth plate (A) Growth environment containing solid media plates with growing mutant Symbiodiniaceae colonies. (B) Example of grown *Breviolum minutum* colonies after 180 s of UV exposure and allowed to grow on solid agar media. Scale bar: 10 mm.^1^ (C) Example of a UV exposure survival curve after analysis.^1^ Error bars shown represent the standard deviation of the percent survival of replicates from the same time exposure. (D) An example of a pigment mutant colony among other mutant colonies with natural variation. Scale bar: 1 mm.
12. Grow colonies for approximately 1–3 months. An example of fully grown colonies on a agar plate is given in Figure 3B.

**CRITICAL:** It is important to check the solid cultures periodically over time to prevent mutant colonies from growing into one another, preventing accurate quantification, and increasing the difficulty of downstream isolation of individual colonies. Colonies can range in size but are ready to be counted when the cells of each colony on the plate are consistent in dark brown in color.

### Survival curve generation

**Timing: 1 day**

Generate a survival curve relating the dosage dependent response of the mutagenized cells to UV exposure period through the quantification of colonies and subsequent plotting the data as a scatter plot in spreadsheet software. A survival curve from Jinkerson et al. 2022 is given as an example in Figure 3C.

13. Using a dissection microscope, count and record the number of surviving colonies for each replicate plate.
14. Group the data based on the same number of mutagenized cells initially plated (1 × 10^5^, 1 × 10^4,^ or 1 × 10^3^ mutagenized cells) for direct comparison.
15. Within each group (1 × 10^5^, 1 × 10^4^, or 1 × 10^3^ mutagenized cells), divide the colony count value of each UV exposure period plate by the colony count value of the unexposed plate of the same replicate number (“r1”, etc.). and multiply the resultant fraction by 100, to obtain the relative percentage of survival at the given UV exposure period.
16. Obtain the average and standard deviation for each resultant percent value.
17. For each group, generate a scatter plot displaying the UV exposure period against percent survival. Change the Y-axis to log scale to observe linearity.

### Mutant screening and isolation

**Timing: 1 month**

Once the survival curve is established, a UV exposure period can be selected based on survival rate and individual experimental needs for the creation of mutants. Grown colonies from the chosen UV exposure period can be isolated and used in downstream processes, such as phenotypic screening. In practice, this can be broken down into two steps: a primary screen to identify possible mutants and a secondary screen to confirm the desired phenotype. Plates of colonies with a UV exposure period correlating with 1% or lower survival have been used successfully for discovery of mutant strains of interest.^1^

18. Choose a phenotypic trait of interest.
19. Visually inspect the colonies corresponding to the UV exposure period of choice for phenotypes of interest using a dissection microscope. An example of a colony with a differing color phenotype is given on Figure 3D.
20. Isolate mutant colonies of interest from solid media growth plates.
  a. In a sterile environment, prepare a separate solid media plate or 96 well plate containing 200 μL of MB based liquid media per well for transfer. The media formulation chosen should be permissible for mutant strain growth.
  b. In a sterile environment, remove the lid of the plate containing the colony of interest and transfer cells from the colony to the destination plate using a sterilized toothpick. Visually ensure cells have securely attached to its surface and that no off-target colony cells have been touched.
  c. Streak the picked cells to fresh solid media or inoculate liquid media for growth and storage.
  d. Allow the isolated mutant cells at least 1 month, in a permissive condition, to grow before use in other applications to ensure recovery and viability of the mutant strain cells.
21. Perform a secondary screen to verify phenotype of interest.
  a. Create multiple backup copies of suspected mutant strains. Potential mutants plated on solid media should be transferred and spread to 2–3 individual solid media plates using sterile toothpicks. For strains suspended in liquid media, transfer 30 μL of the previous culture in a single well into a new well containing fresh 170 μL. These strains should be maintained by transferring to new media monthly for best health.
  b. Place the putative mutant and original *Breviolum minutum* strain into comparable conditions that highlight suspected phenotypic differences and allow growth for approximately 1 month.
  c. Observe the resulting phenotypes of the mutant strains to verify expected phenotypic change.
  d. Continue to propagate mutant strains with validated changes in phenotype compared to the original strain.

***Note:*** Cell colonies can vary in size and have a range of brown coloring due to natural variation, light to darker brown depending on the age and health of the cells in the colony. If performing a secondary screen on solid media, it is important to place the original Symbiodiniaceae strain on the same plate as small variations in the microenvironment may lead to variations in phenotypes.

***Note:*** If necessary, repeat the UV mutagenesis process at the desired exposure period and further plan environmental conditions to highlight the phenotype of interest, including nutrients in the solid media, lighting conditions, and temperature. For example, mutants without the ability to perform photosynthesis were found by growing mutagenized cells heterotrophically in the dark with dextrose.^1^ In practice, approximately 1000 surviving colonies per plate help ensure successful isolation by reducing the chance of accidental contact with off-target colonies.

## Expected outcomes

After the plating of mutagenized cells and placement into their growth environment, colonies will begin to form slowly over the course of 1–2 months but can take longer. Starting colonies may appear to be translucent in the early stages of colony formation but grow to become darker in color over time. Plating density of mutagenized colonies can affect the final size of individual colonies, with lower densities leading to larger sizes.

The phenotypes of isolated mutants can be explored to discover insights into the biology of Symbiodiniaceae and their symbiotic partners. For example, mutants can be added directly to infect host organisms to observe the way in which the mutation affects the symbiosis. This has been previously done with mutants unable to perform photosynthesis, showing that photosynthetic ability is not required for association.^1^

Although the mutations created are random, it may be possible to link phenotype to genotype. Sequencing of genomic and/or transcriptomic sequences can reveal changes in the genome caused by the UV radiation, such as insertions, deletions, or single nucleotide polymorphisms. These changes in genotype may provide molecular insights into the observed phenotypes. Depending on the level of mutagenesis, 10s–100s of mutations may be present. One strategy to narrow potential causative mutations is to isolate multiple mutants sharing the same phenotype and determine genes that have mutations in all the isolates.

## Limitations

The efficacy of germicidal UV lamps depends on multiple factors like strength, age, and brand. Although the exposure times outlined have been used to create a survival curve here and generate mutants, these time exposures may need adjustment based on germicidal lamp availability and efficacy.

## Troubleshooting

### Problem 1

There may be residual liquid present in Petri dishes containing solid media caused by trapped condensation from the agar solidification process. Condensed liquid may encounter mutant colonies on top of the agar media and lead to the unintentional movement of cells within the plate, potentially affecting the reliability of strain isolation.

### Potential solution

• Before transferring liquid agar media into their Petri dishes, ensure the media is as cool as possible without solidifying.
• The solid media Petri dishes containing condensed water can be dried before the transfer of mutagenized cells by placing it in the flow hood or biosafety cabinet with the lid open.
• Closed Petri dishes can be kept in the sterile biosafety cabinet laminar flow hood for approx. 12 h with continuing airflow to help prevent condensation within the lid.

### Problem 2

Close or touching colonies can negatively affect one’s ability to efficiently isolate mutant colonies of interest.

### Potential solution

• Glass pipettes can be used to craft a fine point utensil for isolating colonies. Heat two glass pipettes together with a flame to melt the glass together and pull them apart while still soft to achieve a very thin end.
• If cells of a colony of interest mix with off target cells during isolation, cells grown from the colony of interest can be recovered by widely streaking the cells picked on to solid media coupled with subsequent subculturing. This works best for easily distinguishable color mutants.
• UV mutagenesis can be repeated, and the cell solution can be diluted further than the previously chosen concentrations for plating to control colony number.

### Problem 3

Colony number per plate for plates derived from untreated cells may be lower than expected. This may be due to variation in the health of algal pre-cultures. The health of liquid pre-cultures is essential for generating the desired number of mutants. If a large fraction of the pre-cultures is already dead even before the UV exposure, the number of colonies will be lower than expected.

### Potential solution

• To ensure healthy cultures, the algal cells on solid media used for the initial inoculation of liquid pre-cultures should be as healthy as possible. This is normally indicated by consistent brown color among colony cells.
• Propagate pre-culture cells 2–3 times by while they are in log phase before usage.
• Perform a viability assay before aliquoting cells for UV exposure.

### Problem 4

Colonies of Symbiodiniaceae growing on the same solid media plates display natural variation in visual phenotype, particularly color. Colonies formed from plated UV mutagenized cells can display visual phenotypes not indicative of a mutation, leading to false positives. Color variation among Symbiodiniaceae colonies can be due to a variety of factors unrelated to mutagenesis, such as a colony’s age and health, the presence of carbon in the media like glucose, and light exposure.

### Potential solution

• Compare mutant colonies of similar size to obtain an understanding of color variation due to the number of cells in the colony. Healthy colonies of the same size should have the same color.
• Visualize colonies from the top and bottom.
• Track potential mutants by marking the plate location and comparing the color over time to surrounding colonies.
• Take note of the frequency of different color variations; high frequency phenotypes are unlikely to be mutants.
• Isolate any potential mutants that may have a phenotype for future verification on solid media.
• The rate of colony formation and development varies from colony to colony. Ensure individual cells within each colony are no longer transparent before color phenotype determination.

### Problem 5

Light exposure plays a role in the observable colors of mutated colonies, affecting the efficiency of color-based screens for pigment mutations. Mutant deficiencies in pigment production may be unobservable when colonies are grown under purely heterotrophic conditions in the dark due to loss of pigment production.^7^ Colony color can also be reduced or “bleached” in response to high intensity light conditions.^14^ Changes can be made in the growth conditions to improve color distinction between colonies.

### Potential solution

• To induce pigment formation in colonies grown under dark heterotrophic conditions, short exposure to low light (5–10 μmol photons m^−2^ s^−1^ of PAR) for approximately 1–2 weeks can improve the color of colonies by inducing pigment production.
• To decrease light intensity and prevent “bleaching” of cell pigments, plates can be covered with a piece white paper to increase shading. Plates can also be stacked up to 3 high to increase shading among those in the stack.

## Resource availability

### Lead contact

Further information and requests for resources and reagents should be directed to and will be fulfilled by the lead contact, Joseph Russo (jruss012@ucr.edu).

### Materials availability

No new materials were generated to perform this protocol.

## Data Availability

This protocol did not generate or analyze new datasets or code.
